# Accuracy and Error Trends of Commercially Available Bat Swing Sensors in Baseball

**DOI:** 10.3390/sports10020021

**Published:** 2022-02-06

**Authors:** Yoshitaka Morishita, Tsutomu Jinji

**Affiliations:** 1 Department of Sports Research, Japan Institute of Sport Sciences, Kita-ku, Tokyo 115-0056, Japan; 2 Department of Health and Sports, Niigata University of Health and Welfare, Niigata 950-3198, Japan; 3 Faculty of Human Development, Kokugakuin University, Kanagawa, Yokohama 225-0003, Japan; jinji.tsutomu@kokugakuin.ac.jp

**Keywords:** hitting, inertial measurement unit, motion capture system, swing assessment, accuracy, validation

## Abstract

In baseball, the swing speed and swing angle of the bat just before ball impact are important to increase the speed and horizontal distance of a batted ball. This study investigated the accuracies and error trends of four commercially available bat sensors to measure these parameters. The hitting motions of seven healthy participants were measured simultaneously using the bat sensors and an optical motion capture system, and the swing speeds and swing angles were compared. The swing speed was measured with high accuracy, as indicated by the high intraclass correlation coefficient (ICC) between the bat sensor and the motion capture system measurements (mean ICC = 0.78). However, the ICC for the swing angle was lower (mean ICC = 0.58) than that of the swing speed for all but one bat sensor, indicating low accuracy. Moreover, in the high swing speed range, the accuracy of the swing speed tended to decrease for three bat sensors, but the trend of the swing angle was different among bat sensors. Significant systematic biases or proportional errors were found for all bat sensors, indicating the possibility of error correction. The sensor used in this study can help to evaluate the differences between players with different competition levels and hitting motions. Coaches need to be cautious in taking measurements of players with high swing speeds and in assessing slight changes within an individual.

## 1. Introduction

In recent years, inertial measurement unit (IMU) sensors have become smaller and more affordable; thus, they are being used by competitors, coaches, and researchers [1]. IMUs and their applications are customized for each sport, and motion feedback can be easily obtained without requiring special knowledge. In baseball, the market contains balls that can measure the number and axis of rotation of pitched ball as well as sensors that measure the stress acting on the elbow joint in the pitching motion [2]. In the case of hitting, wireless IMUs that can be attached to the knob of the bat have been developed [3], and several manufacturers have commercialized IMUs that measure the swing speed and trajectory (swing angle) of the bat.

Prior research has shown that the swing speed and swing angle just before ball impact, which can be measured through a sensor attached to the bat knob, are important factors in increasing the speed and distance of the batted ball [4,5]. Increases in the batted ball velocity and distance have been used as metrics for evaluating hitters in Major League Baseball, because recent statistical analyses have shown that increases in the batted ball velocity and distance lead to an increase in score [6], which has a strong relationship with scoring [7]. Moreover, studies have shown that the swing speed increases at higher levels of competition [8], and players measure these parameters using IMUs while they work toward improving their hitting skills.

Measurement instruments need to be highly accurate to correctly analyze and evaluate the variations in striking techniques within and between individuals. The hitting motion has been widely measured in laboratories using high-speed cameras and optical motion capture systems [9,10,11], and these devices exhibit calibration errors in the order of a few millimeters or lower [3,12,13]. Therefore, in laboratory measurements, even slight differences can be evaluated by confirming the effect of training or by comparing the movements of an individual with those of others. On the other hand, regarding the values measured by IMUs, it has been pointed out that the reliability of the swing speed is high within and between individuals, but the accuracy of the swing angle is low [14]. In addition, a study that used a motion capture system to verify the accuracy of the sensor reported that, although there was a significant correlation between the two measurements, the individual data contained errors [15]. Studies that have validated the accuracy of wireless IMU measurements through video analysis and wire-based sensors have indicated that the swing speed can be measured with high accuracy over a narrow range, but with low accuracy over a wide range [13]. Additionally, a study that examined the accuracy of three IMUs reported a decrease in accuracy above 31.3 m/s for IMUs rated as the most accurate [16]. Therefore, it is currently difficult for competitive athletes to utilize IMUs to further improve their skills. The decrease in measurement accuracy could be attributed to the saturation of the accelerometer built into the sensor near impact [13]. To perform measurements of high accuracy over a wide range of swing speeds, algorithms for processing the acceleration signals must be improved; however, when systematic biases or proportional errors exist in the measurements, they can be corrected to increase the accuracy. Although previous studies have explored the application range of IMUs, they have not revealed the error trends of the parameters being measured. In addition, details regarding the measurement accuracy of parameters other than the swing speed (swing angle) are not available.

This study investigates the trends of swing speed and swing angle errors for four commercially available sensors using an optical motion capture system as the gold standard method and examines the possibility of correcting the errors to verify the effective use of the bat swing sensor in practice at various levels of competition. It was hypothesized that the accuracy of the sensor’s swing speed and swing angle would decrease as the swing speed increased, but that the accuracy could be increased by correcting the measured values. This study clarifies the accuracy of various IMUs available in the market without blinding the product names, thereby potentially aiding general users and players in understanding the precautions to be taken when performing measurements using those sensors and popularizing the effective use of IMUs in practice fields. Furthermore, this would provide a basis on which to consider whether or not to implement IMUs in other striking motion studies.

## 2. Materials and Methods

### 2.1. Participants

Seven people participated in the experiment: three male professional baseball players (age: 21.7 ± 0.5 years, height: 1.79 ± 0.01 m, weight: 81.4 ± 4.6 kg) and four male recreational baseball players (age: 28.5 ± 5.3 years, height: 1.71 ± 0.04 m, weight: 67.0 ± 4.2 kg). The experiments were approved by the ethics review committee of Japan Institute of Sport Sciences (IRB #037). Each participant was briefed on the purpose of the study and measurement details prior to participating in the experiments, and written consent was obtained from all participants.

### 2.2. Procedure

Each participant performed a warm-up of five practice swings and then hit the tossed ball with a bat fitted with an IMU. The toss was thrown underhand by the examiner from the direction of the pitcher toward the participant. The distance between the participant and the examiner was approximately 5 m. A total of four different IMUs were used, all of which could be attached to the knob of the bat (Table 1). Each IMU comprises a built-in accelerometer and gyroscope, but their manufacturer, model, and sample rate were not disclosed by the IMU manufacturer. Each participant hit approximately 5–10 balls when using each IMU. As the purpose of this study was to understand the trends in measurements at various competition levels, the participants were asked to perform not only normal swings, but also varying effort levels and movements. Professional players used the wooden bats (length: 0.85–0.86 m, mass: 0.850–0.904 kg) that each player normally uses, while recreational players used either wooden (length: 0.85 m, mass: 0.880 kg, Asics Corp., Japan) or polyurethane bats (length: 0.83 m, mass: 0.595 kg, Nagase Kenko Corp., Japan). Polyurethane bats were used because the wooden bats are too heavy for recreational players to ensure a natural swing. Each IMU is designed so that only one can be attached to the bat, and they were attached to the bat using a special mounting device according to the manufacturer’s instructions. The bat information was input into the dedicated application software (Table 1) downloaded to the tablet, and the bat swing was measured by the software.

**Figure 1:**
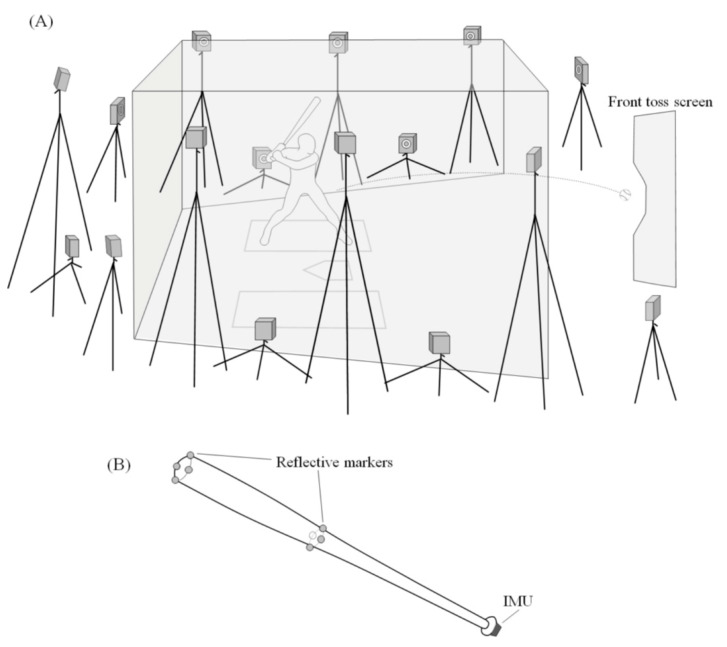
Experimental setup. (**A**) Schematic of the position of 16 optical cameras. (**B**) Location of the reflective markers. The reflective markers at the tip and middle of the bat were placed concentrically and were evenly spaced, respectively.

To verify the accuracy of each IMU, an optical motion capture system (Vicon-Vantage, Vicon Motion Systems Ltd., Oxford, UK; hereinafter, referred to as “Mocap”) consisting of 16 dedicated cameras was used to measure the motion of the bat at 500 Hz. The average 3D reconstruction error (RMS distance in camera pixels) for each camera by Mocap’s dynamic calibration was 1.04 mm. Mocap and IMU measured each trial simultaneously without synchronization. Eight hemispherical reflective markers (diameter: 16 mm) were attached to the bat (Figure 1). The reference coordinate system was defined as the origin of the corner on the catcher’s side of the home plate; the *Y*-axis, as the direction of the pitcher from the origin; the *Z*-axis, as the vertically upward direction; and the *X*-axis, as the direction of the first base perpendicular to both axes. 

### 2.3. Data Processing

The analysis did not use representative values for each participant and all trials were analyzed independently. The parameters measured by the IMUs (swing speed and swing angle) were also calculated from the 3D coordinates of the reflective markers measured by Mocap. The swing speeds in the Swing Tracer (MZ) and Swing Coach (GA) devices were determined based on the assumed velocity of the bat head, as described in the accompanying manual, and the displacement of the bat head in the three frames just before impact. The swing angle was also calculated as the angle between the velocity vector of the bat head and the horizontal plane. The swing speeds in Blast Baseball (BM) and Swing Tracker (DK) devices were calculated from the displacement of the coordinates of a position 0.15 m away from the bat head in the direction of the knob, as in previous studies [13]. The swing angle was also calculated from the velocity vector at the same position; this means that the method of calculating the measured values of BM and DK will be different from that of MZ and GA. The time of ball impact in the motion capture system was set to the frame just before the sudden decrease in the speed of the bat head.

### 2.4. Statistical Analysis

To evaluate the reliability of the measured values of each IMU, the Pearson’s product-moment correlation coefficient and the intraclass correlation coefficient (ICC) were calculated for the swing speed and swing angle measured from each IMU and Mocap [17]. ICC used the two-way random effect model (ICC 2,1) to represent the reliability between the devices when measured with IMU and Mocap for the same attempt [18]. Bland–Altman analyses were conducted to calculate the systematic bias and precision of each IMU using the values measured using Mocap as the true values [19]. Because the differences between the two sensors (BM and DK) and the Mocap data were not normally distributed in this study, logarithmic (natural) transformation of the data was performed before calculating the systematic bias and precision. The data were presented after performing antilog. For the logarithmic transformation, the negative values of the swing angle were addressed by adding 90° to all swing angle values. To investigate whether the systematic bias, which is the difference between the measured value of each IMU and the measured value of Mocap, varies from 0, a one-sample t-test was conducted for each systematic bias [20]. In addition, the presence of the proportional error was confirmed using Pearson’s test. To test the hypothesis that the swing angle is affected by swing speed, a scatter plot of swing speed and swing angle (difference between sensor and Mocap) was created. The significance level for all analyses was set to less than 5%. To determine whether commercially available sensors are effective, the analytical goals regarding reliability were set with reference to a previous study that examined the variability of hitting motion within individuals. Therefore, the analytical goals were set to a total error (systematic bias and random error) not exceeding ± 0.6 m/s and ± 3° for swing speed and swing angle, respectively.

## 3. Results

**Figure 2:**
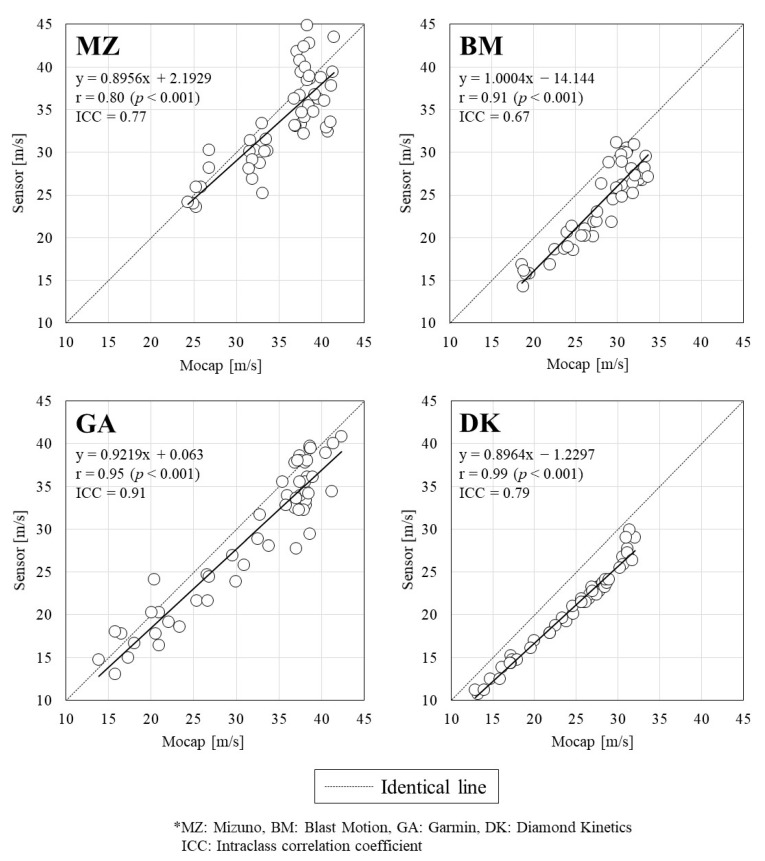
Relationship between swing speeds measured by the motion capture system and the four commercially available sensors.

**Figure 3:**
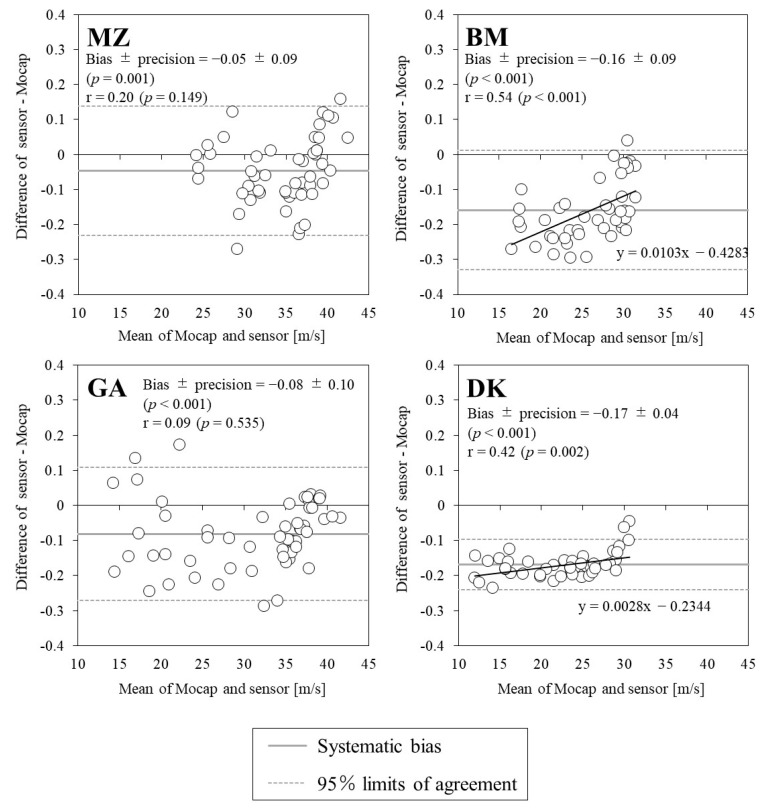
Bland–Altman plots showing the relationships between swing speeds measured using the motion capture system and the four sensors.

Figure 2 illustrates the relationship between the swing speed measurements obtained using the IMUs and Mocap. All of the IMU measurements showed significant positive correlations with the Mocap measurements, and all correlation coefficients were above 0.8. The highest and lowest ICC were observed for GA (=0.91) and BM (=0.67), respectively. Figure 3 shows the Bland–Altman plot for the swing speed of each IMU and Mocap, and Table 2 shows the mean swing speed, systematic bias, and 95% limit of agreement for both devices. The swing speed was found to be a significant systematic bias in all IMU measurements, and the values of all IMUs were estimated to be smaller than those of Mocap. The precision (standard deviation of the difference between methods) of each IMU averaged to 8%, with DK showing the lowest value. The proportional errors showed a significant positive correlation for BM and DK (BM: *p* < 0.001, DK: *p* = 0.002). For IMUs other than GA, it was observed that the random error was larger at a high swing speed (Figure 3).

**Figure 4:**
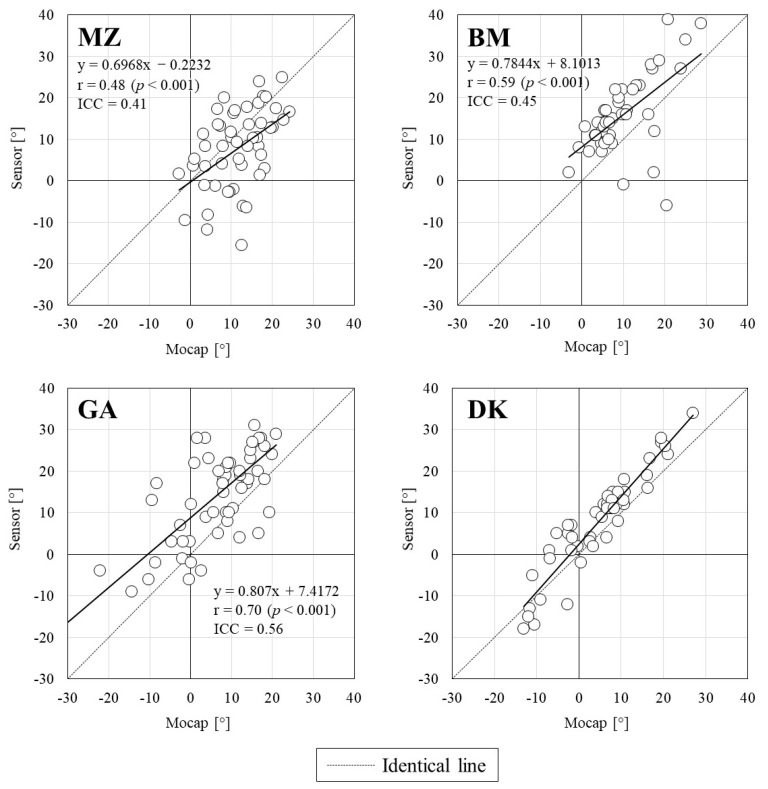
Relationships between swing angles measured using the motion capture system and the sensors.

**Figure 5:**
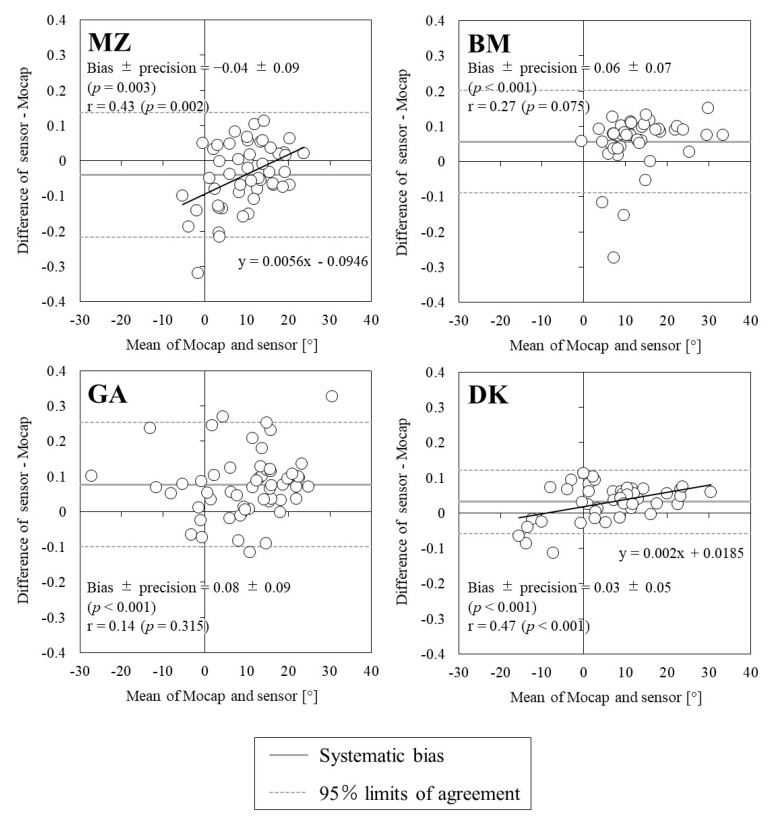
Bland–Altman plots comparing the swing angle measurements obtained using the motion capture system and the sensors.

**Figure 6:**
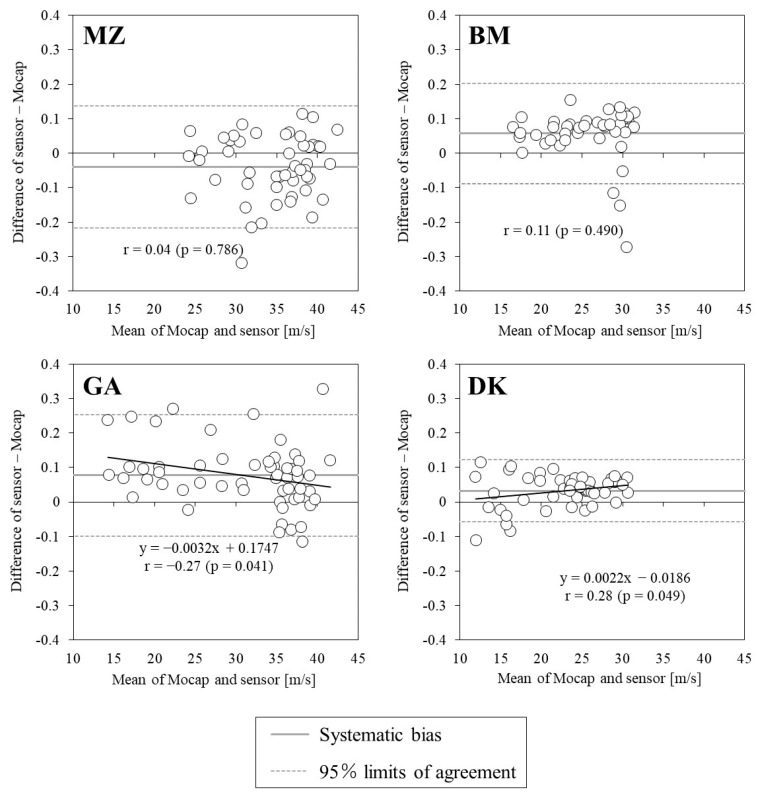
Relationship between swing speed and swing angle measurements obtained using the sensors and the motion capture system.

Figure 4 illustrates the relationship between the swing angles measured using the IMUs and Mocap. All IMU measurements showed significant positive correlations with those of Mocap; however, the correlation coefficients were below 0.8, except for DK. For ICC, DK showed high values (>0.8), but others were below 0.6. Figure 5 shows the Bland–Altman plot for the swing angle of each IMU and Mocap, and Table 3 shows the mean value, systematic bias, and 95% limit of agreement for the swing angles of both devices. The swing angle was a significant systematic bias in all IMU measurements, and the precision was lowest for DK at 5%. The proportional errors were significantly positively correlated with those of MZ and DK (MZ: *p* = 0.002, DK: *p* < 0.001). In the distribution of the swing angle error against swing speed, significant proportional errors were found for GA and DK (Figure 6). For BM and GA, there were several trials in which the swing angle exceeded the 95% limit of agreement in the region of high swing speed, but for DK, there were trials in which the swing angle exceeded the 95% limit of agreement in the region of low swing speed.

## 4. Discussion

The purpose of this study was to examine the accuracy and error trends of IMUs attached to commercially available baseball bats using a motion capture system, and to determine if the errors could be corrected. The hypothesis of this study was that the accuracy of the IMUs’ swing speed and swing angle decreases as the swing speed increases, but that the accuracy could be increased by correcting the measurements. The results showed that all IMUs estimated swing speed less effectively than Mocap, and some IMUs were less accurate for high swing speed. The correlation coefficient between IMUs and Mocap for swing angle was smaller than that for swing speed. In addition, the relationship between swing speed and swing angle showed different trends among IMUs. All IMUs showed significant systematic or proportional errors, indicating that the measured values could be corrected, thus partially supporting the hypothesis of this study.

### 4.1. Accuracy of Swing Speed and Swing Angle

A study examining the bat swing characteristics at various strike points reported that the swing speed varied by up to approximately 1.2 m/s depending on the hitting point [21]. Based on these results, a precision of at least half of 1.2 m/s (i.e., 0.6 m/s) is required to clarify the variability of the swing speed at different hitting points within individuals. In addition, differences of up to approximately 3 m/s in maximum effort swing speed were observed within individuals in the professional baseball players participating in this study. In other words, if the IMUs can perform measurements with a precision of ±1.5 m/s or less, the differences in swing speeds corresponding to the maximum effort exerted by a skilled batter can be determined. The results also indicated that MZ and GA could measure swing speeds in the range of 15–40 m/s, BM and DK in the range of 10–35 m/s, and all IMUs have a moderate or high correlation with the Mocap measurements. In the Mocap measurements in this study, the speeds were calculated at the tip of the bat and at the position 0.15 m below the barrel, and the difference was approximately 5 m/s. Commercially available bat sensors have a similar range of swing speeds; therefore, these sensors can be used to examine speed differences between players at different levels of competition. However, the precision of the swing speed of MZ, BM, and GA was more than 8% (Table 2), suggesting that these IMUs are not able to investigate slight variations within an individual.

The swing angle showed lower agreement with the Mocap measurement (ICC) than the swing speed. The swing angle can vary by up to approximately 6° depending on the hitting point [21]. Therefore, to examine the intraindividual variability of the swing angle at various hitting points, the precision should be within 3°. However, the precision of all the IMUs used in this study was greater than 4% (Table 3); this is not enough to analyze the differences in the swing angle at the same hitting point within an individual or between players with similar swing trajectories.

### 4.2. Error Causes and Trends

The correlation coefficient for swing speed was over 0.8 and ICC was over 0.6 for all IMUs, indicating that the IMUs can measure swing speed with high accuracy for players of various competition levels. However, there was a systematic bias that was smaller than the Mocap swing speed for all IMUs. The random error tended to increase in the region of high swing speed (Figure 3). These results are similar to those reported in a previous study [13] and may be attributed to the saturation caused by exceeding the measurement range of the accelerometer. It was found that swing angle has a smaller correlation coefficient and ICC than swing speed and is difficult to measure with the same accuracy as swing speed. In addition, the trend of systematic bias differed depending on the IMU. The hypothesis predicted the swing angle error to increase with the swing speed; however, the hypothesis was only supported for BM (Figure 6). This factor may be attributed to differences in accelerometer data processing and gyroscopic sensors among the IMUs. In summary, the IMUs can accurately measure swing speed over a wide range, but caution should be exercised when evaluating players whose swing speed exceeds 30 m/s. In a single measurement, all IMUs are not accurate enough to analyze the intraindividual changes in both parameters; therefore, the average value of multiple measurements should be used when evaluating the hitting motion in practice [15].

In contrast, DK was the most accurate among the IMUs used in this study, showing correlation coefficients above 0.9 for both parameters with the Mocap measurements. This strong correlation may be explained by the different attachment devices used for fixing the IMUs to the bat. The attachment device used for DK was made of plastic and could be firmly fixed to the knob, whereas those for the other sensors were made of rubber. The rubber attachments are less secure to the knob than plastic attachments and can shift out of position when the hands make contact with them during each swing. In the experiments conducted in this study, the attachments were examined after each swing to check if they were displaced from the knob. However, minor misalignments cannot be ruled out. Therefore, the differences in the accuracies of the IMUs may have resulted from the differences in the attachments and their positional deviations rather than from the sensitivity or measurement range of the IMUs. IMUs other than DK may be capable of more accurate measurements if fixed firmly to the knob by taping in addition to the attachment. In this study, the IMUs were fixed to the bat according to the product manual; therefore, further investigation is needed to determine the measurement accuracy of the IMU itself and the effect of the method of fixing the IMU to the bat.

In addition, the Bland–Altman analysis of this study confirmed that there are bias and proportional errors in the measured values of each IMU (Figure 3 and Figure 5). By taking these errors into account and correcting the measured values, it is possible to achieve more accurate measurement than the current situation without using Mocap. Sensor technology is evolving continuously, and improved accuracy may be realized in a few years. In the future, the built-in accelerometers and data processing algorithms used in sensors will be enhanced to measure variability within athletes and individuals at higher levels of competition.

### 4.3. Limitations

There are a few limitations to this study. As mentioned in the methodology, the participants were asked to perform varying effort and movement trials as well as normal trials. This was to increase the sample size and to analyze the validity of each IMU on a dataset for players of different competition levels. The analysis did not use representative values for each participant and all trials were included in the analysis, resulting in a sample size of approximately 50 trials per IMU. It cannot be denied that the data contain some bias, but as each participant was allowed to perform the trials in various motions, the effect would be minor. In addition, the study also included data from two different types of bats. It cannot be denied that the different bat materials may have affected the measurements. However, as the speed of vibration transmitted through materials is generally faster than the speed of sound (about 340 m/s), the effect of differences between bats on the measured values is considered to be negligible. Furthermore, to reduce participant fatigue, the IMUs and the motion capture system did not verify the reproducibility of the measurements. For the motion capture system, the calibration error was approximately 1 mm. Thus, it can be inferred that the repeatability of the measurements is high. However, it cannot be ruled out that the reproducibility of the measurements of each IMU may be low. A previous study reported that the IMU reproduces swing speed well, but not the swing angle [14]. Therefore, the tendency of the error in the swing angle shown in this study requires further investigation.

## 5. Conclusions

The accuracies of swing speed and swing angle measured using four commercially available IMUs were examined using Mocap as the gold standard, and the trends in measurement errors were identified. Mocap was selected as the gold standard because it is used in biomechanics research as a reliable instrument that can accurately measure the movement of objects. The results of all IMUs showed significant positive correlations with those recorded using Mocap. However, three of four IMUs exhibited reduced measurement accuracy over higher ranges of swing speeds. The ICC of the swing angle was lower than that of the swing speed, except for one IMU. These findings demonstrate that commercial IMU sensors are useful for evaluating differences in swing speeds among amateur players; however, they may not be suitable for professional players. In addition, whereas swing angle may exhibit a certain tendency if evaluated between athletes with significantly different forms, it would be difficult to investigate variations within an individual regardless of the skill level. Variations in swing angle may exhibit a certain trend if evaluated between players with considerably different hitting styles, but it would be difficult to examine slight variations within individuals regardless of their skill level. Because systematic biases or proportional errors were observed in all of the IMUs when measuring both parameters, compensating for these errors could enable performing measurements with slightly higher accuracies than those obtained currently.

## Figures and Tables

**Table 1:** Commercial names, manufacturers, version of application software, and abbreviations used in the paper for the bat swing sensors used in this study.

**Commercial Name**	Swing Tracer	Blast Baseball	Swing Coach	Swing Tracker
**Manufacturer**	Mizuno	Blast Motion	Garmin	Diamond Kinetics
**Version**	1.4.0	5.4.0	1.0	5.0.0
**Abbreviated Name**	MZ	BM	GA	DK

**Table 2:** Reliability of swing speeds obtained from the motion capture system and the four commercially available sensors (bias and limits of agreement are presented on a ratio scale after antilog).

	Sensor (m/s)	Mocap (m/s)	Bias ± Precision (Ratio)	95% Limits of Agreement (Ratio)
**MZ**	33.9 ± 5.5	35.4 ± 4.9	−0.05 ± 0.09	−0.23 to 0.14
**BM**	24.2 ± 4.9	28.1 ± 4.4	−0.16 ± 0.09	−0.33 to 0.01
**GA**	29.7 ± 8.0	32.1 ± 8.2	−0.08 ± 0.10	−0.27 to 0.11
**DK**	20.9 ± 5.0	24.7 ± 5.5	−0.17 ± 0.04	−0.24 to −0.10

* MZ: Mizuno, BM: Blast Motion, GA: Garmin, DK: Diamond Kinetics.

**Table 3:** Reliability of swing angles obtained from the motion capture system and the four commercially available sensors (bias and limits of agreement are presented on a ratio scale after antilog).

	Sensor (m/s)	Mocap (m/s)	Bias ± Precision (Ratio)	95% Limits of Agreement (Ratio)
**MZ**	7.8 ± 9.5	11.6 ± 6.5	−0.04 ± 0.09	−0.22 to 0.14
**BM**	15.8 ± 9.1	9.8 ± 6.9	0.06 ± 0.07	−0.09 to 0.20
**GA**	14.0 ± 12.6	6.2 ± 10.5	0.08 ± 0.09	−0.10 to 0.25
**DK**	8.2 ± 11.4	4.9 ± 9.3	0.03 ± 0.05	−0.06 to 0.12

## Data Availability

The data presented in this study are available on request from the corresponding author.
